# Interleukin-8 and depressive responses to an inflammatory challenge: secondary analysis of a randomized controlled trial

**DOI:** 10.1038/s41598-022-16364-3

**Published:** 2022-07-24

**Authors:** Jennifer L. Kruse, Chloe C. Boyle, Richard Olmstead, Elizabeth C. Breen, Susannah J. Tye, Naomi I. Eisenberger, Michael R. Irwin

**Affiliations:** 1 Norman Cousins Center for Psychoneuroimmunology, Los Angeles, USA; 2 Department of Psychiatry and Biobehavioral Sciences, David Geffen School of Medicine, Jane and Terry Semel Institute for Neuroscience and Human Behavior at UCLA, 300 UCLA Medical Plaza, Room 2273, Los Angeles, CA 90095 USA; 3 Queensland Brain Institute, The University of Queensland, Brisbane, Australia; 4 Department of Psychiatry & Psychology, Mayo Clinic, Rochester, USA; 5 Department of Psychiatry, University of Minnesota, Minneapolis, USA; 6 Department of Psychology, University of California Los Angeles, Los Angeles, USA

**Keywords:** Interleukins, Predictive markers, Depression, Experimental models of disease, Translational research, Randomized controlled trials

## Abstract

Emerging evidence suggests that interleukin (IL)-8 has a protective role in the context of depression. Higher levels of IL-8 are associated with lower depressive symptom severity among depressed patients, and treatment-related increases in IL-8 correlate with a positive response in depressed patients. This study (a secondary analysis of a completed randomized controlled trial) aimed to examine whether higher levels of IL-8 mitigate increases in depressed mood in response to an experimental model of inflammation induced depression. Given epidemiologic relationships identified between IL-6, tumor necrosis factor (TNF)- α, and subsequent depression, levels of these pro-inflammatory cytokines were also explored as potential moderators of depressed mood response to endotoxin. Secondary analyses were completed on data from healthy adults (*n* = 114) who completed a double-blind, placebo-controlled randomized trial in which participants were randomly assigned to receive either a single infusion of low-dose endotoxin (derived from Escherichia coli; 0.8 ng/kg of body weight) or placebo (same volume of 0.9% saline). IL-8, as well as IL-6 and TNF- α, were measured at baseline prior to infusion, and depressed mood and feelings of social disconnection were assessed approximately hourly. Baseline levels of IL-8, but not IL-6 or TNF-α, moderated depressed mood (*β* = − 0.274, *p* = .03) and feelings of social disconnection (*β* = − 0.307, *p* = .01) responses, such that higher baseline IL-8 was associated with less increase in depressed mood and feelings of social disconnection in the endotoxin, but not placebo, condition. IL-8 had threshold effects, in which highest quartile IL-8 (≥ 2.7 pg/mL) attenuated increases in depressed mood in response to endotoxin as compared to lower IL-8 quartiles (*p* = .02). These findings suggest that IL-8 may be a biological factor that mitigates risk of inflammation-associated depression.

Clinical trials registration: ClinicalTrials.gov NCT01671150, registration date 23/08/2012.

## Introduction

Epidemiologic studies demonstrate that a naturalistic inflammatory challenge (e.g., severe infection) increases the risk of subsequent depression^1^, with further evidence that quasi-experimentally induced inflammation (e.g. interferon alpha treatment; vaccine administration) also elicits depressive symptoms^2,3^. In an experimental model of depression^4^, administration of low-dose endotoxin leads to increases in depressed mood and feelings of social disconnection^4,5^. Moreover, we have found that known risk factors for depression^6^, including female sex, sleep disturbance, and anxiety, as well as peripheral transcriptome profiles related to immune activation, sympathetic activation, and glucocorticoid insensitivity, moderate a heightened vulnerability to depressed mood in response to inflammatory challenge and were associated with greater increases in depressed mood^7,8^. However, less is known about what biological factors might mitigate inflammation-induced depression.

In observational studies, we have found higher levels of the pro-inflammatory cytokine and chemokine interleukin (IL)-8 are related to lower depressive and anxious symptom severity among patients with treatment resistant depression^9^. In addition, when levels of IL-8 increase across a course of treatment with electroconvulsive therapy^10^ or ketamine^11^, these increasing levels of IL-8 are associated with treatment responses in females. No experimental studies have tested whether higher levels of IL-8 might mitigate depressed mood response to an inflammatory challenge.

Given epidemiologic research identifying relationships between pro-inflammatory markers and subsequent risk for depressive symptoms^12–14^, we also explored whether higher baseline levels of IL-6 or TNF-α would predispose healthy participants to greater depressed mood response to an inflammatory challenge.

To address these questions, we completed secondary analyses of data from a completed randomized controlled trial examining the effects of low-dose endotoxin vs. placebo on depressed mood and feelings of social disconnection, in a large sample of healthy adults. We tested whether baseline levels of IL-8 moderate the magnitude of increase in depressed mood and feelings of social disconnection in response to endotoxin versus placebo. We hypothesized that higher IL-8 levels at baseline would be protective with regard to subsequent inflammation-induced depressed mood and feelings of social disconnection. In addition, we explored whether baseline levels of the proinflammatory cytokines IL-6 and TNF-α were associated with greater increase in depressed mood following endotoxin.

## Materials and methods

### Participants

One hundred and fifteen healthy participants completed a randomized study of endotoxin vs. placebo administration between March 2011 and 2013, as described previously^15^. All procedures were approved by the UCLA Institutional Review Board and all research was performed in accordance with relevant guidelines and regulations. Written informed consent was obtained from all participants. Inclusion and exclusion criteria were previously described. For the current study, one participant was excluded due to missing data (missing POMS at T2) resulting in 114 participants (age range 18–50 years; 69 females and 45 males) who were deemed eligible, were randomized, and received endotoxin (*n* = 60) or placebo (*n* = 54) (Table 1).

**Table 1:** Baseline characteristics of the sample variable.

	Placebo (*n* = 54)	Endotoxin (*n* = 60)	Group Differences (placebo vs. endotoxin)
Age, mean (SD)	23.3 (6.0)	25.0 (7.1)	*p* = 0.17
Sex, female, %	57%	63%	*p* = 0.52
**Race**			
White, *n* (%)	25 (46)	19 (32)	*p* = 0.27
Asian/Pacific Islander, *n* (%)	15 (28)	17 (28)	
Latinx, *n* (%)	8 (15)	17 (28)	
Other, *n* (%)	6 (11)	7 (12)	
Body mass index, mean (SD)	23.5 (2.6)	24.3 (2.9)	*p* = 0.15
Baseline POMS depression (mean)	0.09 (0.24)	0.07 (0.22)	*p* = 0.51
Baseline social disconnection	1.9 (0.4)	1.7 (0.5)	*p* = 0.08
Baseline IL-8, pg/mL; median (Q1–Q3)*	1.8 (1.2–2.7)	1.6 (1.0 -2.5)	*p* = 0.65
Baseline IL-6, pg/mL; median (Q1–Q3)*	1.6 (1.2–2.9)	1.6 (1–2.6)	*p* = 0.61
Baseline TNF-α, pg/mL; median (Q1–Q3)*	6.7 (5.8–8.1)	6.2 (5.2–7.1)	*p* = 0.04

*Values were transformed by natural logarithm before all statistical analyses, but original scale medians and IQR are presented here.

### Procedure

Study procedures for this double-blind, placebo-controlled, randomized clinical trial (NCT01671150; 23/08/2012, date of registration on clinicaltrials.gov) have been reported in detail^7,8,15^. Partial results previously reported^7,8,15,16^, include studies on socio-behavioral and transcriptomic predictors of depressed mood response to endotoxin^7,8^. No prior study has examined whether baseline levels of inflammation, including IL-8, are related to increase in depressed mood in response to endotoxin.

Each participant was randomly assigned to receive either an infusion of low dose endotoxin (0.8 ng/kg body weight, Escherichia coli group O:113; BB-IND129487 to MRI) as provided by the National Institutes of Health Clinical Center or placebo (same volume of 0.9% saline). This dose of endotoxin mimics increases in inflammation as found in inflammatory disorders, infections, and psychological stress^17–19^. Circulating levels of IL-8, as well as IL-6 and TNF-α, were measured at baseline (T0, approximately 10 min before infusion) and then approximately every hour post-administration over 6 h (i.e., T1 to T6). Depressed mood, feelings of social disconnection, and physical sickness symptoms were assessed approximately hourly from T0 to T6. All study procedures were approved by the University of California (UCLA) Institutional Review Board.

### Measures

Change in depressed mood from T0 to T2 was assessed using the depression subscale of the short-form Profile of Mood States (POMS)^20,21^. For this scale, participants rate the extent to which they feel: ‘unhappy’, ‘sad’, blue’, ‘hopeless’, ‘discouraged’, ‘miserable’, ‘helpless’, and ‘worthless’ on a scale from 0 (not at all) to 4 (extremely). Measures of depressed mood were calculated by averaging scores from each of these items at each timepoint. The T2 timepoint was selected because endotoxin induced peak increases in depressed mood and feelings of social disconnection at approximately two hours after exposure in this randomized controlled trial^15,16^; thus, baseline moderator analyses would have the greatest power to detect effects on change from the baseline to two hour time point.

Change in feelings of social disconnection from T0 to T2 was assessed using a questionnaire that included 12 items, previously described^15,16^. Similar to depressed mood response, the T2 timepoint was selected because endotoxin induced peak increases in feelings of social disconnection at approximately two hours after exposure^15,16^, and scores were averaged at each timepoint.

#### Moderators of outcome

Whole blood samples were collected in prechilled EDTA tubes. After collection, the samples were centrifuged at 4 °C, plasma was harvested into multiple aliquots, and then stored at − 70 °C until immunoassays were performed.

Data for baseline (T0) levels of IL-8, IL-6, and TNF-α were obtained by high sensitivity bead-based multiplex (Luminex) immunoassays (Performance High Sensitivity Human Cytokine, R&D Systems, Minneapolis, MN, USA), as previously described^15^. Due to the strength of the original study design, which utilized up to seven repeated measures of cytokine values to profile inflammatory cytokine responses for each subject, the plasma sample from each time point was evaluated in a single determination. The average intra-assay CV% for standards was 4.3, 4.4, and 5.3 for IL-6, IL-8, and TNF-α, respectively; the inter-assay CV% of an internal laboratory quality control sample was < 13.5% for all three analytes. The lower limit of detection for IL-8 was 0.1 and values lower than this limit were entered as 0.05 (1/2 the lower limit); *n* = 6 samples).

### Data analysis

All data were examined for distributional qualities. Because baseline cytokine concentrations were not normally distributed, we performed a natural logarithmic transformation on the data prior to statistical analyses. Five baseline cytokine values [one for IL-8 analyses (value of 45.2 pg/mL), one for IL-6 analyses (value of 26.4 pg/mL), and three for TNF-α analyses (values > 44 pg/mL)] were excluded for having concentrations more than three standard deviations above the mean.

Relationships between baseline cytokine concentration, demographics (age, sex, body mass index, race/ethnicity) and baseline behavioral measures of interest (POMS depression score, social disconnection score) were evaluated with bivariate correlations or t-tests to determine whether they should be included as covariates in regression models evaluating relationships between baseline cytokine values and change in behavioral measures. Age was the only variable which demonstrated a significant relationship with baseline IL-8 concentration and was thus included in IL-8 statistical models as a covariate. Sex demonstrated a significant relationship with baseline TNF-α concentration and was included in TNF-α statistical models as a covariate. Models were not otherwise adjusted for covariates, as the study was a randomized, placebo-controlled experimental trial.

To establish between-group differences in the association between baseline IL-8 concentration and behavioral symptom change in response to endotoxin versus placebo, we used multiple regression models with baseline IL-8, condition (0 = placebo; 1 = endotoxin), age, and the baseline IL-8 by condition interaction as independent variables on the dependent variables POMS depression and social disconnection change scores (T2 minus baseline score). Based on substantial evidence that threshold levels of inflammatory biomarkers, such as C-reactive protein (CRP), predict de novo depression onset^22^, response to depression treatment^23,24^, and other health outcomes^25^, multiple regression analyses were repeated using a threshold level of IL-8, as defined by the upper quartile of baseline IL-8 quartile vs. the lower three quartiles (Table 2). Quartiles were utilized for these analyses based on prior research^25,26^ as precise thresholds with potential clinical relevance have yet to be established for IL-8. All analyses were then repeated using baseline IL-6 and TNF-α as moderators.

**Table 2:** Baseline characteristics of the sample stratified by baseline IL-8 quartile.

Variable	Quartile 1 (*n* = 29)	Quartile 2 (*n* = 28)	Quartile 3 (*n* = 28)	Quartile 4 (*n* = 28)
Baseline IL-8, pg/mL; range*	0.05–1.1	1.1–1.7	1.7–2.6	2.7–6.5
Age, mean (SD)	22.2 (3.6)	24.8 (7.5)	23.1 (3.7)	26.6 (9.3)
Sex, female, %	59%	75%	50%	57%
**Race**				
White, *n* (%)	11 (38)	14 (50)	10 (35)	9 (32)
Asian/Pacific Islander, n (%)	5 (17)	7 (25)	12 (43)	8 (29)
Latinx, *n* (%)	8 (28)	5 (18)	4 (14)	7 (25)
Other, *n* (%)	5 (17)	2 (7)	2 (7)	4 (14)
Body mass index, mean (SD)	23.7 (3.2)	24.0 (2.5)	23.6 (2.7)	24.1 (2.7)
Baseline POMS depression	0.1 (0.2)	0.2 (0.4)	0.1 (0.2)	0.1 (0.1)
Baseline social disconnection	1.8 (0.5)	1.8 (0.4)	1.7 (0.5)	1.8 (0.4)
Baseline IL-6, pg/mL; range*	0.5–9.2	0.4–10.9	0.3–9.4	0.3–5.9
Baseline TNF-α, pg/mL; range*	2.9–9.4	2.5–10.8	3.7–10.4	2.1–10.7

*Values were transformed by natural logarithm before all statistical analyses, but raw data are presented here.

To evaluate whether the quartile IL-8 thresholds corresponded with empirically derived IL-8 thresholds, sensitivity analyses were then conducted in which we calculated the effect size of the condition (endotoxin vs. placebo) effect for each individual value of IL-8 across the full range of baseline levels of IL-8. These sensitivity analyses allowed us to estimate the level of IL-8 below which the endotoxin (vs. placebo) condition effect on depressive symptoms and social disconnection was no longer significant.

Additional sensitivity analyses were conducted controlling for presence of physical sickness symptoms at T2; controlling for physical sickness symptoms did not meaningfully alter results (data not shown).

Analyses were conducted using IBM SPSS (Version 27) and Stata SE/16.1. Statistical significance was established at *p* ≤ 0.05 (two-tailed) and results are presented as standardized beta coefficients (*β*), with the semi-partial correlation squared (sr^2^) provided as a standard measure of effect size (with 0.01, 09, 0.25 indicating small, medium, and large effects^27^.

The primary analysis was the evaluation of baseline IL-8 in relation to endotoxin-induced POMS depression change. Given remaining analyses represented exploratory efforts to identify possible mechanisms for future study, corrections for multiple testing were not applied.

### Sample size determination/power analysis

As a secondary analysis of a completed randomized clinical trial, the sample size was set by other outcomes, but previously observed main and moderated effects of IL-8 on depressive symptoms were large and would be detectable within this sample size with power ≥ 80%.

## Results

### Baseline characteristics

Table 1 summarizes participant demographic information and baseline concentrations of cytokines. Demographic characteristics, baseline behavioral variables, and baseline levels of IL-8 and IL-6 did not differ between the endotoxin and placebo groups, although level of TNF-α did differ by group (see Table 1). Baseline IL-8 concentration was significantly related to age (*r* = 0.19, *p* = 0.04) and all analyses with IL-8 thus included age as a covariate. Consistent with this, beyond age there were similarly no other baseline group differences as a function of baseline IL-8 quartile (see Table 2). Baseline TNF-α concentration was lower in females as compared to males, *t*(108) = 3.11, *p* = 0.002, so analyses with TNF-α controlled for sex. IL-6 was not related to any baseline characteristic. Unadjusted analyses were not substantively different from adjusted analyses.

### Baseline IL-8 as a moderator of endotoxin-induced change in depressed mood

**Figure 1:**
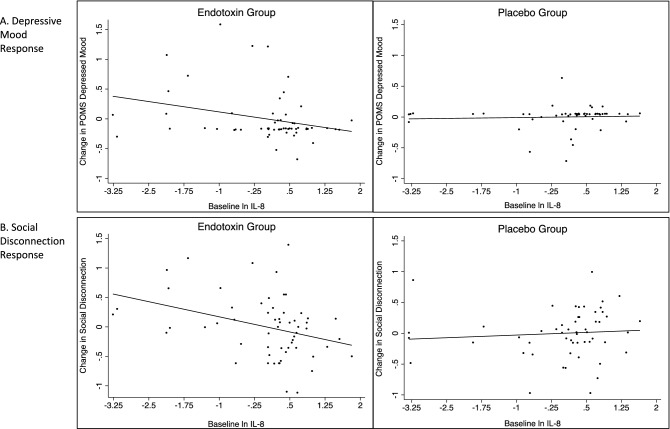
Association between baseline IL-8 and change in depressive mood (**A**) and social disconnection (**B**) Baseline ln IL-8 was significantly associated with depressive mood response and social disconnection response within the endotoxin group (*p*’s < .03) but not the placebo group (*p*’s > .69). Analyses are adjusted for age. See Table 1S for full statistical results.

There was a significant baseline IL-8 by condition (endotoxin vs. placebo) interaction for POMS depression change from baseline to T2 (approximately 2 h following endotoxin or placebo infusion), controlling for age, (β = -0.274, *p* = 0.03), such that higher baseline IL-8 was associated with less increase in depressed mood among endotoxin-exposed participants (*β* = − 0.304, *p* = 0.03; effect size (sr^2^) = 0.09) but not placebo-exposed participants (*β* = 0.056, *p* = 0.69; effect size (sr^2^) = 0.003) (see Fig. 1A).

**Figure 2:**
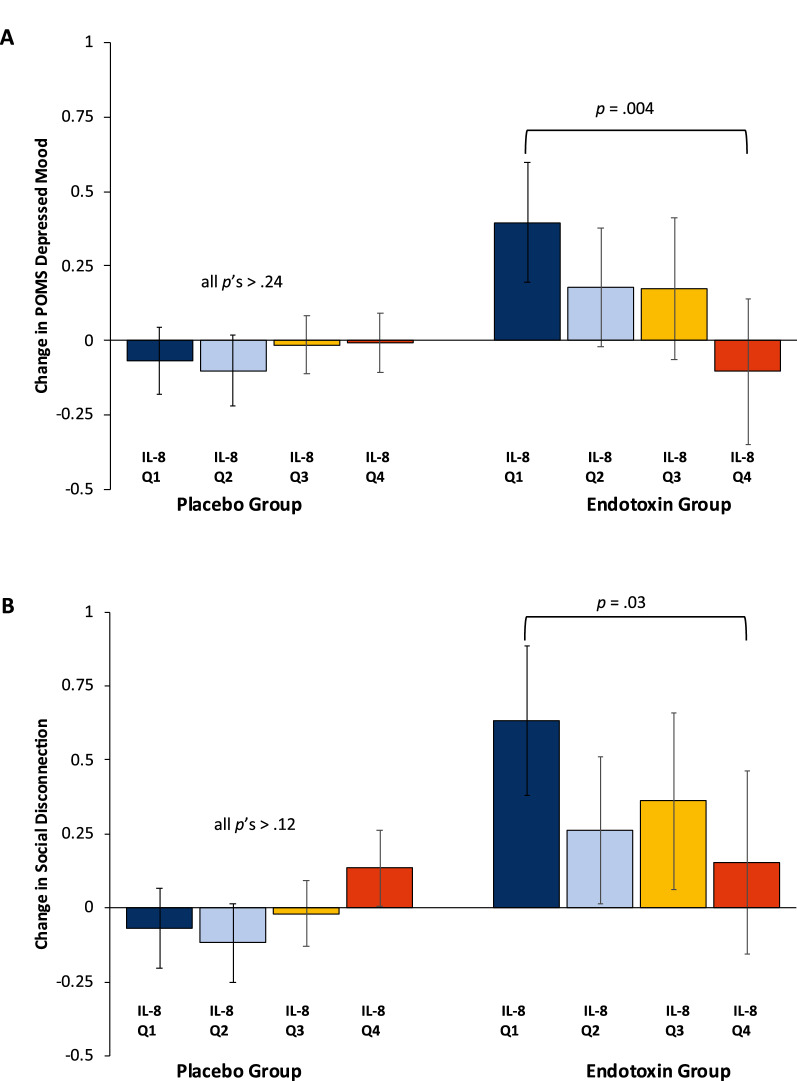
Predictive means for change in behavioral measures by baseline IL-8 quartile Within the endotoxin group, the upper IL-8 quartile (Q4) showed lower depressive mood response as compared to the lowest IL-8 endotoxin quartile (Q1) (*p* = .004) and the lower three IL-8 quartiles combined (Q4 to Q1-3, *p* = .02), but not individually (Q4 to Q2*, p* = .08; Q4 to Q3, *p* = .12). (**A**). The Q4 endotoxin quartile also showed lower social disconnection response as compared to Q1 (*p* = .03) but not all three combined (Q4 to Q1-Q3, *p* = .15) or individually (Q4 to Q2, *p* = .59; Q4 to Q3, *p* = .35). (**B**). Error bars are 95% Confidence Intervals.

To test for a potential threshold effect, we evaluated baseline IL-8 quartile as an independent variable for POMS depression change from baseline to T2. Multiple regression analysis demonstrated a significant baseline IL-8 quartile x condition interaction for POMS depression change, controlling for age (*β* = − 0.665, *p* = 0.004). In follow-up contrast analyses within the endotoxin group, endotoxin-exposed participants in the upper IL-8 quartile (≥ 2.7 pg/mL) had significantly lower mean POMS depression change as compared to endotoxin-exposed participants in the lowest IL-8 quartile (≤ 1.1 pg/mL; *p* = 0.004) and as compared to endotoxin-exposed participants in the three lower IL-8 quartiles combined (*p* = 0.02). There was no relationship between baseline IL-8 and POMS depression change in the placebo condition (all *p*’s > 0.24). See Fig. 2.

**Figure 3:**
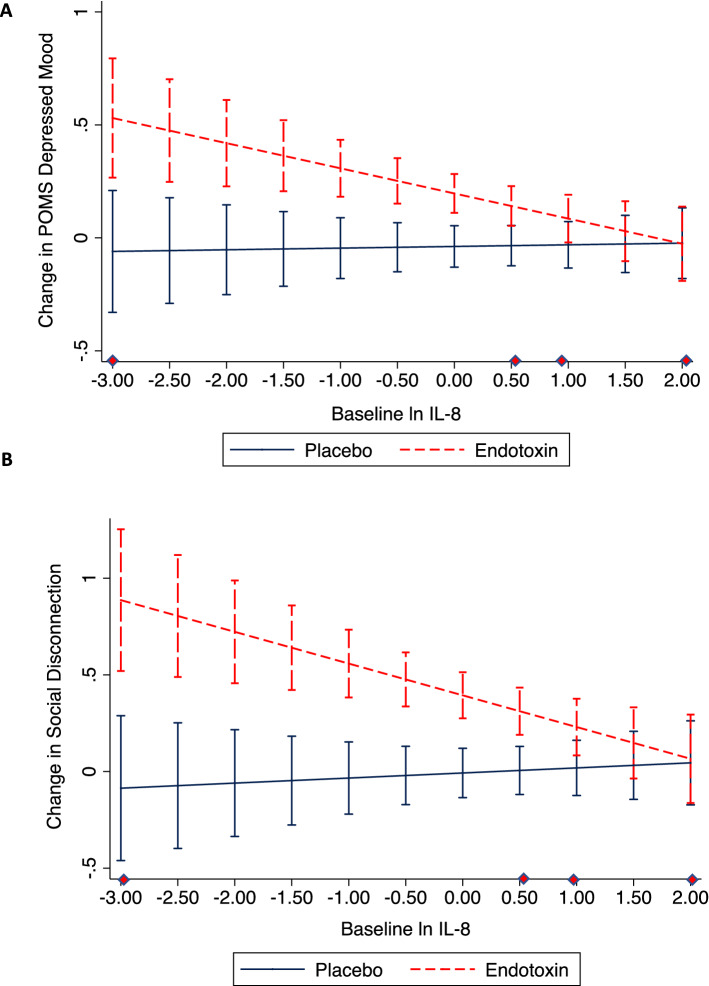
Sensitivity analyses: Endotoxin vs. control group differences in behavioral symptom change across the range of baseline IL-8 concentrations. Across baseline levels of IL-8 in the endotoxin vs. control group, there is a greater increase in depressed mood (**A**) and social disconnection (**B**) up until the threshold of ln IL-8 = 0.90 (antilogarithm 2.5 pg/mL) (**A**) and ln IL-8 = 1.05 (antilogarithm 2.9 pg/mL) (**B**), which roughly corresponds with the cut-off for the upper IL-8 quartile (2.7 pg/mL). Error bars are 95% Confidence Intervals. Diamond symbols demarcate baseline IL-8 quartiles.

To test for an empirically derived threshold of IL-8 at which endotoxin no longer elicited significantly greater increases in depressed mood relative to placebo, sensitivity analyses were conducted to examine the simple effect of condition (endotoxin vs. placebo) on POMS depression change from baseline to T2, across the full range of baseline levels of IL-8. As illustrated in Fig. 3a, this threshold roughly corresponded with the cutoff for defining the upper quartile of baseline IL-8 (i.e., ≥ 2.7 pg/mL).

### Baseline IL-8 as a moderator of endotoxin-induced change in feelings of social disconnection

As expected, changes in depressed mood and social disconnection from T0 to T2 were moderately correlated (*r* = 0.58, *p* < 0.001). There was a significant baseline IL-8 by condition interaction for social disconnection change (*β* = − 0.307, *p* = 0.01), controlling for age, such that higher baseline IL-8 was associated with reduced change in social disconnection among endotoxin-exposed participants (*β* = − 0.364, *p* = 0.007; effect size (sr^2^) = 0.12) but not placebo-exposed participants (*β* = 0.085, *p* = 0.55; effect size (sr^2^) = 0.007) (see Fig. 1B).

As above, a threshold effect of IL-8 on change in social disconnection was tested and showed a significant baseline IL-8 quartile x condition interaction (*β* = − 0.565, *p* = 0.01). Follow-up contrast analyses demonstrated that endotoxin-exposed participants in the upper IL-8 quartile had significantly lower mean social disconnection change as compared to endotoxin-exposed participants in the lowest IL-8 quartile (*p* = 0.03) but not as compared to endotoxin-exposed participants in the lower three IL-8 quartiles combined (*p* = 0.15). There was no relationship between IL-8 and social disconnection change in the placebo condition (all *p*’s > 0.12; see Fig. 2b).

Sensitivity analyses also evaluated an empirically derived threshold of IL-8, and demonstrated this threshold roughly corresponded with the cutoff for defining the upper quartile of baseline IL-8 (Fig. 3b).

### Baseline IL-6 and TNF-α as moderators of endotoxin-induced changes in depressed mood and feelings of social disconnection

The baseline IL-6 by condition interactions for depressed mood change (*p* = 0.21) and social disconnection change (*p* = 0.91) were not significant. Similarly, the baseline TNF-α by condition interactions were non-significant for change in depressed mood (*p* = 0.58) and social disconnection (*p* = 0.95), adjusted for sex. There was also no indication for threshold effects as a function of baseline IL-6 or TNF-α quartiles (all *p*’s > 0.08).

## Discussion

This report provides initial data supportive of a protective role for IL-8 in mitigating inflammation-induced depressed mood. In association with higher IL-8 levels at baseline, the magnitude of increase in depressed mood in response to endotoxin was attenuated. Further, there was a threshold effect, such that the uppermost IL-8 quartile was associated with significantly lower mean depression change as compared to IL-8 values below this threshold. Baseline IL-8 also attenuated increases in feelings of social disconnection in response to inflammatory challenge. Findings that higher baseline IL-8 levels were associated with less subsequent increase in depressed mood or feelings of social disconnection, in response to inflammatory challenge, were of medium effect size, suggesting potential clinical relevance of IL-8 in mitigating depressive symptom onset in the setting of inflammation. In contrast, baseline levels of the cytokines IL-6 and TNF-α were not related to subsequent inflammation-induced depressed mood or feelings of social disconnection.

The current IL-8 findings complement previous findings in depressed patients, in which higher IL-8 was associated with lower severity of depressive symptoms^9^, and increasing IL-8 levels over a course of depression treatment (ECT or ketamine) were associated with improving depression symptoms among females^10,11^. Together, these converging results suggest that the inflammatory marker IL-8 may be protective against the onset of depressive symptoms and may reduce the severity of depressive symptoms. Conversely stated, a “deficit” of IL-8 may be a vulnerability factor for the development of depressive symptoms, and/or associated with greater symptom severity. While the mechanisms underlying these relationships remain unclear, IL-8 may have neuroprotective^28–30^ and neurotrophic^31^ properties and plays an important role in angiogenesis and maintenance of endothelial integrity through upregulation of vascular endothelial growth factor (VEGF)^32^. Pre-clinical and translational work are needed to further test the behavioral effects of IL-8 and to elucidate mechanisms that may underlie the observed associations between IL-8 levels and depressive symptoms in humans.

Given prior work demonstrating that greater baseline activation of the transcription factor NF-kB is associated with greater increase in depressed mood in response to endotoxin^8^, one might expect an association between higher baseline levels of the inflammatory cytokines IL-6 and TNF-α and greater depressed mood increase, since NF-kB upregulates production of these cytokines. Likewise, lower levels of these pro-inflammatory markers might be hypothesized to be associated with a resilient phenotype (to the extent that lower levels serve as an indirect marker of a system under less physiological or psychological distress.) However, no such relationships were identified in the current study. It is most plausible that the increased activation of NF-kB represents an increased state of “readiness” for responsiveness to an inflammatory challenge (such as endotoxin) with downstream effects on circulating pro-inflammatory cytokines. It is the subsequent inflammation-associated increase in proinflammatory cytokines that is associated with the development of depressed mood and feelings of social disconnection in response to endotoxin^15^. Moreover, simply having lower levels of certain circulating pro-inflammatory markers may not be sufficient to actively confer protective effects against inflammatory challenge.

While the randomized, placebo-controlled design is a major strength of the current study, interpretations regarding the behavioral effects of IL-8 remain limited, given that IL-8 levels themselves were not independently manipulated in the current study. Direct manipulation of IL-8 levels would be ideal for testing causal relationships with behavioral symptoms. In addition, the absence of central measures of inflammation (e.g. cerebrospinal fluid concentrations of IL-8) is a limitation of the current study. Future studies of inflammation-associated behavioral symptoms may benefit from analysis of cerebrospinal fluid levels of IL-8, and/or neuroimaging measures of central inflammation. Further, future studies with larger sample sizes and more comprehensive behavioral phenotyping may provide the opportunity for increased granularity regarding whether effects of baseline IL-8 have more specificity with regard to impacts on particular behavioral measures. Another important contextual feature of the current study is that the participants were healthy and without history of psychiatric illness. The extent to which inflammation-induced behavioral symptoms in a non-psychiatric population are relevant to clinical psychiatric populations is unclear. Similarly, the behavioral measures evaluated in the current study do not clearly reflect full depressive symptom responses over time, but rather acute responses specifically related to depressed mood and feelings of social disconnection. However, the study of such variably-induced symptoms in healthy populations is a critical step towards understanding potential underlying mechanisms of symptom development, including potential protective and vulnerability factors, which may inform future efforts to prevent or treat inflammation-associated behavioral symptoms.

To our knowledge, this is the first study evaluating and identifying a protective effect of baseline IL-8 on depressed mood increase in response to an experimentally-induced inflammatory challenge. Given converging findings across varying populations, there is compelling evidence to suggest that IL-8 may be protective against the development of depressive symptoms and also may reduce the severity of mood symptoms among depressed patients. With this possibility comes an urgent need for deeper interrogation of potential behavioral and biological mechanisms underlying the identified associations with IL-8, such that these findings may ultimately translate into improvements in patient care.

## Supplementary Information

Supplementary Information.

## References

[1] Benros ME, Waltoft BL, Nordentoft M, Ostergaard SD, Eaton WW, Krogh J (2013). Autoimmune diseases and severe infections as risk factors for mood disorders: A nationwide study. JAMA Psychiat..

[2] McNutt MD, Liu S, Manatunga A, Royster EB, Raison CL, Woolwine BJ (2012). Neurobehavioral effects of interferon-alpha in patients with hepatitis-C: symptom dimensions and responsiveness to paroxetine. Neuropsychopharmacology.

[3] Harrison NA, Brydon L, Walker C, Gray MA, Steptoe A, Critchley HD (2009). Inflammation causes mood changes through alterations in subgenual cingulate activity and mesolimbic connectivity. Biol. Psychiat..

[4] Lasselin J, Lekander M, Benson S, Schedlowski M, Engler H (2020). Sick for science: experimental endotoxemia as a translational tool to develop and test new therapies for inflammation-associated depression. Mol. Psychiatry.

[5] Eisenberger NI, Inagaki TK, Mashal NM, Irwin MR (2010). Inflammation and social experience: An inflammatory challenge induces feelings of social disconnection in addition to depressed mood. Brain Behav. Immun..

[6] Slavich GM, Sacher J (2019). Stress, sex hormones, inflammation, and major depressive disorder: Extending social signal transduction theory of depression to account for sex differences in mood disorders. Psychopharmacology.

[7] Irwin MR, Cole S, Olmstead R, Breen EC, Cho JJ, Moieni M (2019). Moderators for depressed mood and systemic and transcriptional inflammatory responses: A randomized controlled trial of endotoxin. Neuropsychopharmacology.

[8] Cho JH, Irwin MR, Eisenberger NI, Lamkin DM, Cole SW (2019). Transcriptomic predictors of inflammation-induced depressed mood. Neuropsychopharmacology.

[9] Kruse JL, Olmstead R, Hellemann G, Breen EC, Tye SJ, Brooks JO (2021). Interleukin-8 and lower severity of depression in females, but not males, with treatment-resistant depression. J. Psychiatr. Res..

[10] Kruse JL, Olmstead R, Hellemann G, Wade B, Jiang J, Vasavada MM (2020). Inflammation and depression treatment response to electroconvulsive therapy: Sex-specific role of interleukin-8. Brain Behav. Immun..

[11] Kruse JL, Vasavada MM, Olmstead R, Hellemann G, Wade B, Breen EC (2021). Depression treatment response to ketamine: Sex-specific role of interleukin-8, but not other inflammatory markers. Transl. Psychiatry.

[12] Beydoun MA, Obhi HK, Weiss J, Canas JA, Beydoun HA, Evans MK (2020). Systemic inflammation is associated with depressive symptoms differentially by sex and race: A longitudinal study of urban adults. Mol. Psychiatry.

[13] Gimeno D, Kivimaki M, Brunner EJ, Elovainio M, De Vogli R, Steptoe A (2009). Associations of C-reactive protein and interleukin-6 with cognitive symptoms of depression: 12-year follow-up of the Whitehall II study. Psychol. Med..

[14] Au B, Smith KJ, Gariepy G, Schmitz N (2015). The longitudinal associations between C-reactive protein and depressive symptoms: Evidence from the English Longitudinal Study of Ageing (ELSA). Int. J. Geriatr. Psychiatry.

[15] Moieni M, Irwin MR, Jevtic I, Olmstead R, Breen EC, Eisenberger NI (2015). Sex differences in depressive and socioemotional responses to an inflammatory challenge: Implications for sex differences in depression. Neuropsychopharmacology.

[16] Moieni M, Irwin MR, Jevtic I, Breen EC, Cho HJ, Arevalo JM (2015). Trait sensitivity to social disconnection enhances pro-inflammatory responses to a randomized controlled trial of endotoxin. Psychoneuroendocrinology.

[17] Ishihara K, Hirano T (2002). IL-6 in autoimmune disease and chronic inflammatory proliferative disease. Cytokine Growth Factor Rev..

[18] Steptoe A, Hamer M, Chida Y (2007). The effects of acute psychological stress on circulating inflammatory factors in humans: a review and meta-analysis. Brain Behav. Immun..

[19] Breen EC, Rezai AR, Nakajima K, Beall GN, Mitsuyasu RT, Hirano T (1990). Infection with HIV is associated with elevated IL-6 levels and production. J. Immunol. (Baltimore, Md. 1950).

[20] Baker F, Denniston M, Zabora J, Polland A, Dudley WN (2002). A POMS short form for cancer patients: Psychometric and structural evaluation. Psychooncology.

[21] McNair, D.M., Lorr, M., Droppleman, L.F. EITS manual for the Profile of Mood States. San Diego, CA: Educational and Industrial Testing Service; 1971.

[22] Pasco JA, Nicholson GC, Williams LJ, Jacka FN, Henry MJ, Kotowicz MA (2010). Association of high-sensitivity C-reactive protein with de novo major depression. Br. J. Psychiat..

[23] Uher R, Tansey KE, Dew T, Maier W, Mors O, Hauser J (2014). An inflammatory biomarker as a differential predictor of outcome of depression treatment with escitalopram and nortriptyline. Am. J. Psychiat..

[24] Raison CL, Rutherford RE, Woolwine BJ, Shuo C, Schettler P, Drake DF (2013). A randomized controlled trial of the tumor necrosis factor antagonist infliximab for treatment-resistant depression: The role of baseline inflammatory biomarkers. JAMA Psychiat..

[25] Ridker PM, Hennekens CH, Buring JE, Rifai N (2000). C-reactive protein and other markers of inflammation in the prediction of cardiovascular disease in women. N. Engl. J. Med..

[26] Jokela M, Virtanen M, Batty GD, Kivimaki M (2016). Inflammation and Specific Symptoms of Depression. JAMA Psychiat..

[27] Cohen J (1988). Statistical Power Analysis for the Behavioral Sciences.

[28] Giovannelli A, Limatola C, Ragozzino D, Mileo AM, Ruggieri A, Ciotti MT (1998). CXC chemokines interleukin-8 (IL-8) and growth-related gene product alpha (GROalpha) modulate Purkinje neuron activity in mouse cerebellum. J. Neuroimmunol..

[29] Puma C, Danik M, Quirion R, Ramon F, Williams S (2001). The chemokine interleukin-8 acutely reduces Ca(2+) currents in identified cholinergic septal neurons expressing CXCR1 and CXCR2 receptor mRNAs. J. Neurochem..

[30] Saas P, Walker PR, Quiquerez A-L, Chalmers DE, Arrighi J-F, Liénard A (2002). A self-defence mechanism of astrocytes against Fas-mediated death involving interleukin-8 and CXCR2. Neuroreport..

[31] Araujo DM, Cotman CW (1993). Trophic effects of interleukin-4, -7 and -8 on hippocampal neuronal cultures: Potential involvement of glial-derived factors. Brain Res..

[32] Martin D, Galisteo R, Gutkind JS (2009). CXCL8/IL8 stimulates vascular endothelial growth factor (VEGF) expression and the autocrine activation of VEGFR2 in endothelial cells by activating NFkappaB through the CBM (Carma3/Bcl10/Malt1) complex. J Biol Chem..

