# Supplementary material for: Interleukin-8 and depressive responses to an inflammatory challenge: secondary analysis of a randomized controlled trial
Source: Sci Rep. 2022 Jul 24;12:12627. doi: 10.1038/s41598-022-16364-3 (PMC9309160; doi:10.1038/s41598-022-16364-3)
Supplement: Supplementary file 1 — Supplementary Information. [file 41598_2022_16364_MOESM1_ESM.docx]

Table 1S. Regression Models: Baseline IL-8 as a Moderator of Endotoxin-Induced Change in Depressed Mood and Social Disconnection

| Outcome | Predictor | *B* | $\beta$ | *SE* | *t* | *p*-value |
| --- | --- | --- | --- | --- | --- | --- |
| Change in Depressed Mood |  |  |  |  |  |  |
|  | Group | 1.877 | .334 | 0.509 | 3.69 | .0004 |
|  | Baseline IL-8 (continuous) | 0.059 | .024 | 0.310 | 0.19 | .850 |
|  | Group X Baseline IL-8 | -0.949 | -.274 | 0.439 | -2.16 | .033 |
|  | Age | 0.056 | .132 | 0.039 | 1.45 | .151 |
|  | Intercept | -1.655 |  | 0.967 | -1.71 | .090 |
| Change in Social Disconnection |  |  |  |  |  |  |
|  | Group | 0.402 | .402 | 0.088 | 4.55 | <.001 |
|  | Baseline IL-8 (continuous) | 0.026 | .059 | 0.054 | 0.49 | .627 |
|  | Group X Baseline IL-8 | -0.190 | -.307 | 0.076 | -2.50 | .014 |
|  | Age | 0.009 | .124 | 0.007 | 1.40 | .165 |
|  | Intercept | -0.234 |  | 0.168 | -1.39 | .166 |

*Note*. Group (0 = placebo; 1 = endotoxin)

*Abbreviations*. IL-8 = interleukin-8.
